# Food-Offering Calls in Wild Golden Lion Tamarins (*Leontopithecus rosalia*): Evidence for Teaching Behavior?

**DOI:** 10.1007/s10764-018-0069-z

**Published:** 2018-11-21

**Authors:** Camille A. Troisi, Will J. E. Hoppitt, Carlos R. Ruiz-Miranda, Kevin N. Laland

**Affiliations:** 1 School of Biology, University of St Andrews, St Andrews, KY16 9TH UK; 2 Present Address: Department of Psychology, University of Cambridge, Cambridge, CB2 3EB UK; 3 School of Biology, University of Leeds, Leeds, LS2 9JT UK; 4 Present Address: School of Biological Sciences, Royal Holloway, University of London, Egham, UK; 5 Laboratory of Environmental Sciences, Universidade Estadual do Norte Fluminense, Campos dos Goytacazes, Rio de Janeiro, Brazil

**Keywords:** Golden lion tamarins, Playback, Primates, Social learning, Teaching, Vocal communication

## Abstract

Many animals emit calls in the presence of food, but researchers do not always know the function of these calls. Evidence suggests that adult golden lion tamarins (*Leontopithecus rosalia*) use food-offering calls to teach juveniles which substrate (i.e., microhabitat) to forage on, or in, for food. However, we do not yet know whether juveniles learn from this aspect of the adults’ behavior. Here we examine whether juveniles learn to associate food-offering calls with a foraging substrate, as a step toward assessing whether these calls qualify as teaching behavior. We compared the performance of four wild juvenile golden lion tamarins that were introduced to a novel substrate while exposed to playbacks of food-offering calls (experimental condition) to the performance of three juveniles that were exposed to the novel substrate without the presence of food-offering playbacks (control condition). We varied the location of the novel substrate between trials. We found that food-offering calls had an immediate effect on juveniles’ interactions with the novel substrate, whether they inserted their hands into the substrate and their eating behavior, and a long-term effect on eating behavior at the substrate. The findings imply that juvenile golden lion tamarins can learn through food-offering calls about the availability of food at a substrate, which is consistent with (but does not prove) teaching in golden lion tamarins through stimulus enhancement. Our findings support the hypothesis that teaching might be more likely to evolve in cooperatively breeding species with complex ecological niches.

**Electronic supplementary material:**

The online version of this article (10.1007/s10764-018-0069-z) contains supplementary material, which is available to authorized users.

## Introduction

Teaching is one of many ways by which individuals transmit knowledge and skills to one another. It is an important process in human development and cultural learning, and was regarded as a uniquely human behavior for a long time (Caro and Hauser 1992; Premack 2007). Recently, however, a few strong cases and many suggestive instances of teaching in nonhuman animals have raised questions about the validity of this assumption (Hoppitt *et al.* 2008; Thornton and Raihani 2008). Caro and Hauser (1992) defined teaching in animals as a behavior that satisfies three criteria: 1) an individual *A* modifies its behavior in the presence of a naïve observer *B*; 2) this modification comes either at a cost or no direct benefit to individual *A*; and 3) individual *B* acquires a new skill or knowledge, or acquires such skill and knowledge sooner than it would otherwise have done. Although there is currently considerable interest in animal teaching, there are, at present only four nonhuman species that fulfill Caro and Hauser’s (1992) functional definition of teaching: tandem-running ants (*Temnothorax albipennis*: Franks and Richardson 2006), meerkats (*Suricata suricatta*: Thornton and McAuliffe 2006), pied babblers (*Turdoides bicolor*: Raihani and Ridley 2008), and superb fairywrens (*Malarus cyaneus*: Colombelli-Négrel *et al.* 2012; Kleindorfer *et al.* 2014a, b).

The centrality of teaching to human behavior and evolution motivate further understanding of the evolution of this costly behavior (Laland 2017a, b). Ethical and logistical issues constrain experimentation on wild animal populations, and partly explain the lack of evidence for teaching in the wild. The majority of experimental studies of social learning and cultural transmission focus on the initial transfer of information between individuals, and more work is required to examine the stability of socially learned behavior over time in natural animal populations (Gunhold *et al.* 2014). The fact that, humans aside, none of the species in which teaching has been demonstrated are primates may seem surprising. However, theoretical work suggests that proficiency in other forms of learning may make it more difficult for teaching to evolve and that the costly and prolonged provisioning often seen in cooperative breeders promotes teaching (Fogarty *et al.* 2011). Evidence for teaching behavior also comes from species where relatedness between teachers and pupils is high, and contexts in which the costs of learning from inadvertent social learning or asocial learning are high (e.g., solitary hunting) (Fogarty *et al.* 2011; Thornton and Raihani 2008).

Callitrichids are an ideal family in which to investigate potential teaching behavior as they are cooperative breeders, potential teachers and pupils are highly related given their social structure, and they hunt individually for prey. They also have a broad diet, which includes fruits, flowers, nectar, exudates, bird eggs, fungi, insects, and small vertebrate prey (Dietz *et al.* 1997; Lapenta *et al.* 2003; Rylands 1989). Their food resources are patchily distributed over large home ranges, and are ephemeral (Dietz *et al.* 1997). This creates the need for learning during ontogeny of what foods are good to eat, where to find them, and how to access them. Teaching could facilitate the acquisition of this information, especially given the short maturation period of callitrichids.

Data from laboratory studies support the view that callitrichids teach one another foraging skills. Knowledgeable adult marmosets and tamarins encourage their young to solve a novel foraging task in experimental studies, which is similar to obtaining food from a novel substrate successfully (Dell’Mour *et al.* 2009; Humle and Snowdon 2008). Adult tamarins also transfer to juveniles foods that are novel to the juveniles more frequently than familiar food, suggesting that the adults modify their behavior to promote juveniles’ learning of diet (Rapaport 1999). Moreover, some callitrichids, such as the red-bellied tamarins (*Saguinus labiatus*) and cotton-top tamarins (*Saguinus oedipus*), transmit information through food calls by increasing their food call rates with the magnitude of their preference for the food (Caine *et al.* 1995; Elowson *et al.* 1991; Roush and Snowdon 2000, 2001).

Consistent with data showing that adult callitrichids modify their behavior to promote learning in juveniles, there is suggestive evidence of teaching in the context of food-offering calls in golden lion tamarins (*Leontopithecus rosalia*: Rapaport 2011). Golden lion tamarins give two types of calls in the presence of food: food calls and food-offering calls (Boinski *et al.* 1994; Brown and Mack 1978; Ruiz-Miranda *et al.* 1999; Ruiz-Miranda and Kleiman 2002). Food calls are high-pitched chattering vocalizations with a peak frequency of about 5 kHz, also called “clucks” (Boinski *et al.* 1994; Brown and Mack 1978). Food calls are usually emitted when an individual sees, or possesses, food (Brown and Mack 1978). If the food call sound is produced on its own in the presence of food (“food call”) then the vocalizer is usually not approached by listeners (Boinski *et al.* 1994). Where the individual emits variable tonal sounds following the food call (“food-offering call”) then another individual usually approaches the vocalizer and takes food with little resistance from the original possessor (Brown and Mack 1978; Ruiz-Miranda *et al.* 1999).

Golden lion tamarins can transmit information about food in two ways. In captivity, golden lion tamarins increase their rate of food calling in line with their preference for a particular food (Benz *et al.* 1992). This suggests that food calls have the potential to transmit information about the individual’s preference and experience with that food. Adult golden lion tamarins also transfer food to other individuals, where the individual with the food does not resist another individual taking its food. Although both adults (mostly pregnant females) and juveniles take food, the majority of food-takers are juveniles between 3 and 9 mo old, and evidence of transfers to adults comes largely from captive or reintroduced individuals (Rapaport 2001; Ruiz-Miranda *et al.* 1999).

The presence of “invitational” signals preceding food transfer distinguish food transfers in golden lion tamarins from other forms of unresisted food taking in primates (Brown and Mack 1978; Rapaport and Brown 2008; Ruiz-Miranda *et al.* 1999). Of those signals, food-associated calls seem to be the most significant in predicting an unresisted food transfer, suggesting that the call transmits information about whether the caller is likely to share food with others (Brown and Mack 1978). In the wild, the use of food-associated calls prior to a transfer has been reported only in transfers from adults to juveniles (Rapaport 2011; Rapaport and Ruiz-Miranda 2006).

Rapaport and Ruiz-Miranda (2002) suggested that adults may also use food-offering calls to indicate the microhabitat feature that is the source of their food (henceforth “substrate”) to their young. This is particularly interesting if juveniles, the usual respondents to those calls, can use the information acquired to find food later in life. In the wild, Rapaport and Ruiz-Miranda (2002, p. 1064) found three instances in which “wild adult golden lion tamarins appear to have directed their immature offspring to a location where a hidden prey item was located”. Rapaport (2011) reports a further 12 instances of this behavior. In these observations, adults were foraging in the absence of visible prey, and emitted calls that attracted a juvenile to the foraging location. The juvenile then started foraging at this location and retrieved prey, and the emitter of the call made no attempt to obtain the prey item (Rapaport and Ruiz-Miranda 2002). These two studies provide evidence for the first of the teaching definition criteria, where adult golden lion tamarins modify the context in which food-offering calls are used. The behavior described by Rapaport and Ruiz-Miranda (2002) and Rapaport (2011) is similar to co-foraging, in which a juvenile approaches and forages on a substrate where another individual is foraging, and no food-offering call is emitted, but co-foraging bouts without a call being emitted by the adult are relatively unsuccessful (Rapaport 2011; Rapaport and Ruiz-Miranda 2002). Thus, it seems that adults emit a call to attract a juvenile to a prey item they have found but not retrieved, suggesting that the behavior functions to teach juveniles where to find food.

Further evidence of adult golden lion tamarins modifying their behavior (i.e., the first criterion of the teaching definition) comes from data showing that as juveniles get older, food-offering calls are used less in a food-transfer context, because juveniles forage more independently, and instead adults start using the calls to direct the juveniles’ attention to a substrate where they can successfully retrieve prey (Rapaport 2011). This supports the hypothesis that adults provide food-related information to juveniles by offering them an opportunity to learn about the substrates on which prey items can be found.

The remaining two criteria of the definition of teaching remain unexplored in golden lion tamarins. However, in terms of the second criterion (i.e., the teaching behavior should incur a cost or no direct benefit), emitting a food-offering call and letting another individual take the prey is likely to be costly, although the magnitude of the cost has not yet been assessed. In the 15 putative teaching cases, the location of the prey varied (crevices in vines, knotholes in branches, broken branch tips, curled dead leaves, tangle of dead leaves and vines), but the prey were always concealed in vegetation (Rapaport 2011). Given that the location of the calls varied between cases, it is likely that juveniles learn from those calls that a particular type of substrate, rather than a specific location, contains food. Prey location varies through the day and across seasons, so learning in which substrate to find prey would be more useful than learning about a particular location. This behavior would qualify as the passing on of general skills, strategies or rules, as opposed to “telling” (Csibra 2007; Leadbeater *et al.* 2006). However, whether the juveniles learn from this modified behavior (the third criteria) remains untested.

We focused on the third criterion of Caro and Hauser’s definition and investigated whether juvenile golden lion tamarins can learn to associate food-offering calls with a novel foraging substrate. Our experiment has two objectives: 1) to discover whether juvenile golden lion tamarins are more likely to interact with a novel foraging substrate, and forage more efficiently, when exposed to experimental manipulations that increase the food-offering call rate than juveniles without this experience; and 2) to assess whether juvenile golden lion tamarins previously exposed to a novel substrate through experimental manipulations that increase the food-offering call rate are more likely to forage from that substrate on reaching independence and whether they do so more efficiently than juveniles without such experience. If juveniles learn from being exposed to food-offering calls, then we predict an increase in foraging success or an increase in foraging effort on the substrate or similar substrates, in juveniles previously exposed to playbacks compared to juveniles that did not experience playbacks.

## Methods

### Design

We compared the performance of wild juvenile golden lion tamarins that were introduced to a novel substrate while exposed to playbacks of food-offering calls (experimental condition) to the performance of juveniles that were exposed to the novel substrate without the presence of food-offering playbacks (control condition). Any behavioral difference between the conditions could be due to an immediate effect of the playbacks (e.g., calls could make the substrate more salient), or to a long-lasting (i.e., learned) behavioral difference that persists over time. We therefore compared animals in the two conditions over two time periods. First, at the time of exposure (February–March 2014), we provided the juveniles with the opportunity to associate the playback calls with the novel foraging substrate (immediate effects). Second, 6 mo later (September–October 2014), we assessed the juveniles’ longer-term learning (long-term effects).

### Subjects

We studied six readily accessible groups of wild golden lion tamarins that were habituated to regular human contact and consistently monitored, in Silva Jardim municipality, Rio de Janeiro, Brazil. Initially there were 10 juveniles across those 6 groups, but 3 disappeared either immediately before the start of the experiment (February 2014) or between the period assessing immediate and long-term effects. Therefore, we included data from 5 groups of 3–10 free-living golden lion tamarins in the analysis (*N* = 35 individuals in total, *N* = 7 juveniles, Table SI of the Electronic Supplementary Material [ESM]). Three groups were at the Poço das Antas Biological Reserve (22°30′–22°33′S; 42°15′–42°19′W), and two groups were in a pocket of Atlantic forest at the Fazenda Afetiva-Jorge, Imbaú region (22°37′S, 42°28′W). The Fazenda Afetiva-Jorge is <30 km from the Poço das Antas Biological Reserve and the two forests have similar plant species (Carvalho *et al.* 2006). We allocated groups randomly to the control or experimental treatment. In two groups all individuals were related to the breeding pair and in three of the groups one individual was not. All individuals were second- or third-generation descendants of reintroduced animals.

Each study group had one or two juveniles (golden lion tamarins often give birth to twins). At the start of the experiment, all juveniles were 5–6 mo old. We chose this age range because juveniles were still dependent on adults for provisioning, and to match previous captive studies (Price and Feistner 1993; Rapaport 1999). The groups were habituated to human presence and monitored by members of the Associação Mico-Leão-Dourado, so the month of birth was known. Each group was captured twice a year, and individuals were weighed, measured and individually marked with Nyanzol dye on the tail and body, and tattooed at birth as part of the management of the species by the Associação Mico-Leão-Dourado (Ruiz-Miranda *et al.* 1999). We assessed long-term effects of food-offering calls on the foraging behavior of juveniles when they were 11–12 mo old.

### Apparatus

**Fig. 1:**
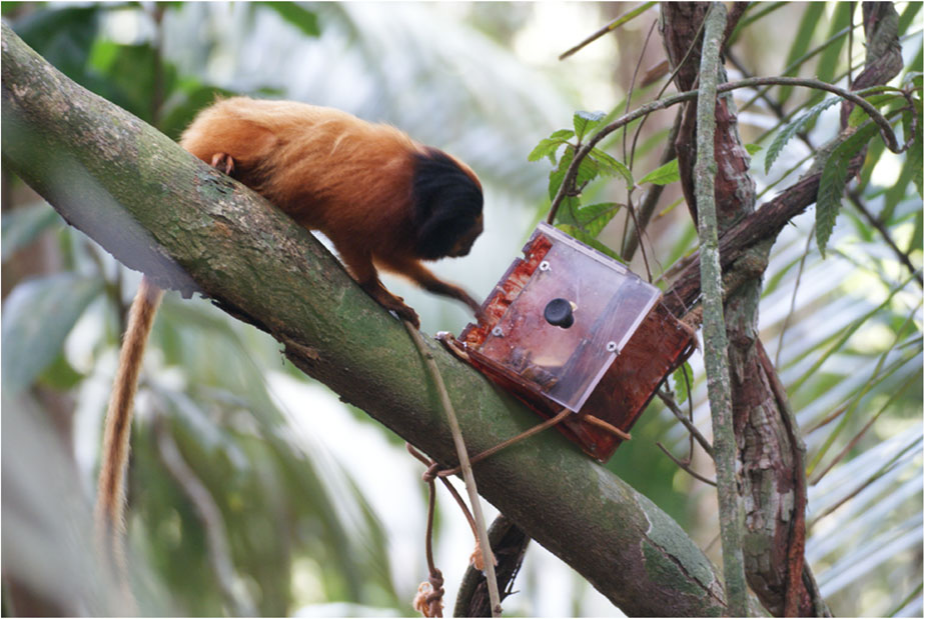
Box attached to a branch used to test whether golden lion tamarins at Poco das Antas and Affetiva learned about substrate properties through food-offering calls, September 2014. The speaker (out of sight) was below the box.

The novel substrate was a cubic plastic box painted brown except the door and top (Fig. 1). The box was similar to that used with vervet monkeys (*Chlorocebus pygerythrus*), but modified so that there was only one option to open the door: sliding it with a handle (van de Waal *et al.* 2013, 2015). We attached the box either to a foraging platform or to branches at human chest level. We used magnets to keep the door open by 1.4 cm. We filled the box with slices of bananas (1–1.5 bananas in each trial) and placed a slice on top of the box to attract the subjects to the box. Bananas were familiar and highly desirable food to monitored groups, and researchers offered them to the groups when seeking to monitor or capture individuals. The opening of the box was wide enough for the subjects to insert their hand into and retrieve food items. We designed the apparatus such that extracting food from it resembled a behavior that golden lion tamarins perform naturally in the wild when they extract prey from narrow holes or cavities, while providing a novel substrate.

We used a Saul Mineroff (SME-AFS) speaker and an ipod mini to emit the sounds for playbacks. The main frequency of the calls was 5–10 kHz, and we used a 5-watt speaker, with a frequency response from 100 Hz to 12 kHz. We placed the speaker under or behind the box and the amplitude of the sounds emitted by the playback calls was around 70 dB, which is in the auditory range of decibels level for golden lion tamarins. We took food-offering calls from prior work on golden lion tamarin vocalizations in the population. The calls were from adults unknown to the animals in our study. We used three different adult calls: one for each of the three groups in the experimental condition. The duration and structure of the calls used in playback were the same as naturally occurring calls. We show one of the calls in the ESM (Fig. S1). We looped a call 10 times to create a stimulus that lasted 5–8 s. Looping calls has previously been used to study both vocalization and behavior of golden lion tamarins, and no behavioral differences have been found between responses to such playbacks and naturally occurring calls (Ruiz-Miranda *et al.* 2002).

### Procedure

#### Assessing Immediate Effects

We assigned two groups to the control condition and three groups to the experimental condition. In the control condition, we exposed groups to a novel foraging substrate. In the experimental condition, in addition to exposing the groups to a novel foraging substrate, we played food-offering calls from the speaker to the subjects at irregular intervals when they were in the vicinity of the box. We played calls when any member of the group was in the author’s visual field. This included individuals approaching and individuals interacting with the box. The rest of the group was in the vicinity of the box.

We presented the box (and playbacks in the experimental condition) to the subjects over five trials. We conducted each trial on a different day, and the location of the box (and playbacks) varied because we conducted the trials whenever we encountered the groups. We tested groups on their own and considered trials invalid if the animals did not interact with the box, if fewer than two individuals (of any age) were at the location of the box for <3 min in total, or if the group taking part in the trial was displaced by another group during the trial. We conducted trials until five valid trials had been completed per group so that each group would have approximately the same opportunities, and we filmed all trials for later analysis. We let the trials continue until all individuals had left and were out of sight and hearing range for a few minutes (average length of trial: 9 min 24 s, standard deviation: 4 min 56 s).

#### Assessing Long-Term Effects

To assess the long-term effects of food-offering calls, we conducted five trials for each group using the same criteria as for the immediate effects. This time, however, we only provided access to the box containing food to all groups. We did not use playbacks.

### Video Analysis

We extracted data from videos using the software package VideoLAN Client (VLC). We recorded behavior patterns (Table I) as states in Microsoft Excel but treated them as events in the analysis. We double coded 10% of the videos and found high interobserver reliability (*r* = 0.80, *P* < 0.001 for all behavior; interaction: *r* = 0.85, *P* < 0.001; insertion: *r* = 0.74, *P* < 0.001; eating: *r* = 0.90, *P* < 0.001).

**Table I:** Definitions of dependent variables used to examine whether wild golden lion tamarins learn substrate properties through food-offering calls. Data collected in 2014, at Poco das Antas and Affetiva, Brazil

Behavior	Definition
Interaction	The individual orients its face toward the box and is close enough to sniff it (no physical contact, but close proximity) or handles the box (requiring physical contact between hands or feet and the box).
Insertion	The individual inserts a hand (or head in some cases) into the box to retrieve bananas. Can be successful (bananas extracted) or not.
Eating	After extracting a food item, the individual ingests it. We counted a maximum of one eating event for each food extraction.

### Statistical Analysis

Our analysis is based on the seven juveniles from five groups present during the two periods, and given this small sample size our findings must be interpreted with caution. We carried out all analyses using R version 3.2.1 (R Development Core Team 2015). We took results with *P* < 0.01 as strong evidence of a difference between the two conditions, and 0.01 < *P* < 0.05 as reasonable evidence. We interpreted 0.05 < *P* < 0.1 as suggestive evidence of a difference due to an unavoidably small sample size (four juveniles in the experimental condition and three in the control). Because null hypothesis testing does not provide the magnitude and the precision of that effect of interest, we also provide effect size statistics and their confidence intervals (CIs) calculated with the *tes* function from the *compute.es* package (Del Re 2013). The effect size estimates the strength of a relationship, while the confidence intervals show the precision of an estimation (Nakagawa and Cuthill 2007). Given the type of data, and the fact that the data were normally distributed in most cases, we used Hedges’ *g* for the effect size for all cases for consistency. Hedge’s *g* is similar to Cohen’s *d*, but is corrected for small sample size (Lakens 2013). Hedges’ *g* provides a scale-free measure of the size of an effect. Cohen (1988) provides a rule of thumb regarding effects to be small (0.2), medium (0.5), and large (0.8), which we follow when verbally reporting effect sizes (Lakens 2013). However, we urge the reader to consider this rule as providing arbitrary points to help judge the magnitude of the effects, and not as hard statistical thresholds. In cases in which we have only suggestive evidence of an effect (0.05 < *P* < 0.1) we expect the 95% CIs to overlap with 0: in these cases we also provide the 90% CIs in the ESM (Table SII).

We inferred learning from the number of successful eating events and compared eating events across conditions to examine the role of food-offering calls in the learning process. Two factors determine the number of successful eating events: 1) the number of insertion events and 2) the probability that insertion successfully retrieves food. Hence, we analyzed those two determining factors first before testing for an overall effect of playbacks on 3) the number of eating events. We also 4) investigated whether the food-offering calls attracted individuals’ attention toward the box by analyzing the number of interactions with the novel substrate.

Initially, we used *t*-tests to compare conditions. However, in some cases, the assumptions of normality were rejected (Shapiro-test; *P* < 0.05) so we present the results from a randomization test using the two-tailed *t*-test statistic, and a null distribution generated by randomizing the data between the conditions 100,000 times. Such randomization tests make no assumptions about the distribution of the data, and have similar power to parametric tests and so are preferable to nonparametric tests (Manly 2006). We used the package *pastecs* to obtain descriptive statistics (Grosjean and Ibanez 2014).

To analyze the probability of insertion events leading to a successful eating event (the number of successful eating events out of the total number of insertions for each individual at each stage of the experiment) we used a *GLMER* with a binomial error structure and logit link function (Bates *et al.* 2015). This is different from analyzing the number of eating events because it looks at variables affecting the probability of an eating event, given that the juvenile has already inserted its hand in the box. It can therefore be considered as a measure of skill or persistence in obtaining food. We added random effects for individuals nested within groups. The main effects were the time period (immediate effects or long-term effects) and whether or not there was playback in the period assessing immediate effects (condition). We used a second *GLMER* to analyze the data on the probability of insertion events leading to a successful eating event, with period and condition as main effects, an interaction between the period and the condition, and random effects for individuals nested within groups. The interaction allows us to test whether there was an effect of playbacks on the difference between periods. We tested the dataset to ensure that the assumptions were not violated. We checked for overdispersion using the dispersion_glmer function in the *blmeco* package (Korner-Nievergelt *et al.* 2015). We used the *AICcmodavg* package to compare the two models using the Akaike information criterion corrected for small sample sizes (AICc)(Mazerolle 2017). AIC is an index that takes into account the likelihood of the model as well as the number of parameters in that model (through parsimony), and ranges between 0 and 1 (Nakagawa and Cuthill 2007). Using AICs avoids issues related to conventional *P* values, such as an arbitrary threshold (Grueber *et al.* 2011). Moreover, by penalizing the model for the number of parameters, this approach minimized the number of falsely positive predictors included in each model (Waite and Campbell 2006).

We also analyzed the adults’ behavior to understand whether the food-offering call had an effect on all individuals rather than just juveniles, and to examine whether a difference in the adults’ behavior could explain any observed differences in the juveniles’ behavior. Using randomization tests, we first analyzed the insertion, eating, and interaction behavior of adults in the control and experimental conditions. We also compared the number of food transfers in the two conditions and the number of food calls emitted by adults in five independent foraging trials. For those independent trials we analyzed both food calls and food-offering calls, as the audio files came from video files, and the quality of the files made the food-offering calls indistinguishable from food calls.

#### Data Availability

The datasets analyzed during the current study and the R Code used to analyze them are available in the open science framework repository and can be assessed at http://osf.io/syexm.

## Ethical Note

We performed the experiment in accordance with the American Society of Primatologists’ (ASP) Principles for the Ethical Treatment of Primates and the Association for the Study of Animal Behaviour (ASAB) guidelines. The Animal Welfare and Ethics Committee of the University of St Andrews approved the study. Instituto Chico Mendes de Conservação da Biodiversidade (ICMBio) approved the ethics for project number 17409–10, “Manejo de metapopulação do mico-leão-dourado: pesquisa e ações,” and the ethics adhered to the legal requirements of Brazil. This species is closely monitored since reintroduction and some individuals that took part in this study had collars for radio-telemetry purposes before the start of the study. However, we did not add any collars for the purpose of this study, nor we did not capture any individuals for this study. The authors declare that they have no conflict of interest.

## Results

### Adult Behavior

We found no significant evidence of an effect of the playbacks on adult foraging behavior, for either period (Table II). When analyzing whether the behavior of adults toward juveniles varied between the two conditions, we found that the provisioned bananas initially in the novel substrate were rarely transferred successfully to juveniles (29 times in total over the two periods for all five groups, 19 in the first period and 10 in the second period, compared to 313 independent eating events from the novel substrate), and we found little evidence of a difference of successful food transfers received by juveniles in the control and experimental condition during this experiment (Table II). We also found no significant evidence for a difference between the number of food-associated calls emitted by adult individuals in the control and experimental conditions during five independent foraging trials (Table II).

**Table II:** Results of randomization tests testing for differences in adult golden lion tamarins’ behavior in groups that were provided with playbacks (experimental condition) and in groups that were not (control condition). Poco das Antas and Affetiva, February–March 2014 (immediate effects) and September–October 2014 (long-term effects)

Behavior	Period	*t*	df	*P* value	g	95% CI
Insertion	Immediate effects	−0.02	22.43	0.990	−0.01	−0.78; 0.76
Insertion	Long-term effects	−0.56	18.61	0.574	−0.01	−0.89; 0.87
Eating	Immediate effects	−0.89	24.88	0.383	−0.33	−1.11; 0.44
Eating	Long-term effects	−0.43	16.49	0.678	−0.18	−1.04; 0.68
Interaction	Immediate effects	1.35	21.40	0.187	0.50	−0.28; 1.28
Interaction	Long-term effects	−0.93	18.17	0.366	−0.38	−1.24; 0.48
Food transfer	Immediate effects	1.43	4.01	0.257	0.92	−0.85; 2.69
Food transfer	Long-term effects	0.15	3.96	1.00	0.10	−1.56; 1.75
Food calls	Independent foraging trials	−0.42	13.16	0.674	−0.19	−1.19; 0.80

*g* refers to the effect size

### Juvenile Behavior

#### Insertion Behavior

**Fig. 2:**
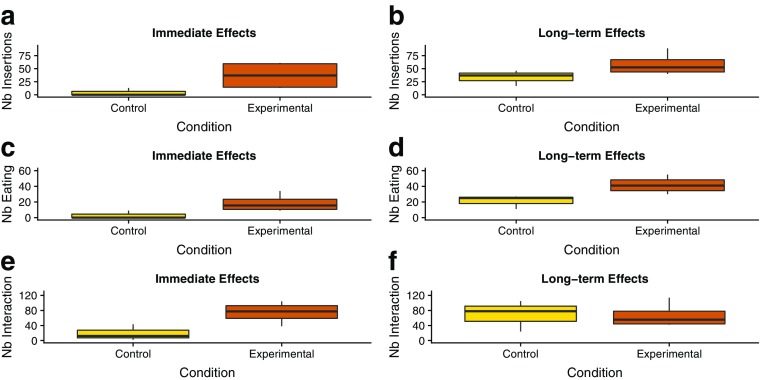
Mean number of insertion events (**a**, **b**), eating events (**c**, **d**), and interaction events (**e**, **f**) in juvenile golden lion tamarins, in groups that were provided with playbacks (experimental condition, orange) and in groups that were not (control condition, yellow). Data collected at Poco das Antas and Affetiva, in February–March 2014 (immediate effects) and September–October 2014 (long-term effects).

When we assessed immediate effects, on average, juveniles in the experimental condition inserted their hands into the novel box more times (M ± SE = 37.00 ± 13.29, *N* = 4) than juveniles in the control condition (M ± SE = 4.33 ± 4.33, *N* = 3). There was suggestive evidence of a difference (Randomization test: *t* = 2.34, df = 3.61, *P* = 0.087; Fig. 2a); with a large effect (*g* = 1.50, 95% CI = −0.45, 3.45).

When we assessed long-term effects, on average, juveniles in the experimental condition inserted their hands into the novel box substrate more times (M ± SE = 58.50 ± 11.02, *N* = 4) than juveniles in the control condition (M ± SE = 33.33 ± 8.57, *N* = 3). This difference was not significant (Randomization test: *t* = 1.80, df = 4.99, *P* = 0.143; see Fig. 2b), but the effect was large (*g* = 1.16, 95% CI = −0.68, 2.99).

#### Probability of a Successful Insertion Event

In a model assuming additive effects of period and condition, we found little evidence of an effect of condition but there was suggestive evidence that there was a higher proportion of successful insertions in the period assessing long-term effects than in the period assessing immediate effects (Table III). When we included an interaction between condition and period, we found little evidence that the effect was stronger in the experimental condition than in the control condition (Table IV). The model without the interaction fit the data better (model without interaction: number of parameters (*K*) = 4, AICc = 84.32; model with interaction: number of parameters (*K*) = 5, AICc = 85.88).

**Table III:** Results of a generalized linear mixed model looking at the proportion of successful insertion events of juvenile golden lion tamarins. Includes period (immediate or long-term effects) and condition (control or experimental) as fixed effects. Data from Poco das Antas and Affetiva, February–March 2014 (immediate effects) and September–October 2014 (long-term effects)

Predictor	Estimate	Std. error	95% CI	*z* value	Pr(>|*z*|)
Intercept^a^	0.107	0.292	−0.68, 0.47	0.367	0.714
Period = assessing long-term effects (relative to the period assessing immediate effects)	0.769	0.213	0.35, 1.19	3.607	<0.001***
Condition = experimental (relative to control)	0.199	0.270	−0.33, 0.73	0.737	0.461

^***^Indicates P values less than 0.001

^a^Baseline was set to training phase and control condition

**Table IV:** Results of generalized linear mixed model looking at the proportion of successful insertion events of juvenile golden lion tamarins. Includes period, condition, and the interaction between the two as fixed effects. Data from Poco das Antas and Affetiva, February–March 2014 (immediate effects) and September–October 2014 (long-term effects)

Predictor	Estimate	Std. error	95% CI	*z* value	Pr(>|*z*|)
Intercept^a^	0.863	0.627	−0.37, 2.09	1.377	0.169
Period = assessing long-term effects (relative to the period assessing immediate effects)	−0.322	0.647	−1.59, 0.95	−0.467	0.619
Condition = experimental (relative to control)	−0.857	0.655	−2.14, 0.43	−1.307	0.191
Interaction: difference in effect of condition (immediate effects – long-term effects)^b^	1.240	0.687	−0.11, 2.59	1.805	0.071

^a^Baseline was set to the period assessing the immediate effects and control condition

^b^Testing whether the difference between the period assessing immediate and long-term effects is larger in the experimental condition than in the control condition

#### Eating Behavior

When we assessed immediate effects, on average, juveniles in the experimental condition ate more times (M ± SE = 18.5 ± 5.69, *N* = 4) than juveniles in the control condition (M ± SE = 3.0 ± 3.0, *N* = 3). There was suggestive evidence of a difference between conditions (Randomization test: *t* = 2.41, df = 4.39, *P* = 0.057, see Fig. 2c), with a large effect (*g* = 1.55, 95% CI = −0.42, 3.52).

When we assessed long-term effects, on average, juveniles in the experimental condition ate more times (M ± SE = 41.74 ± 5.51, *N* = 4) than juveniles in the control condition (M ± SE = 21.0 ± 5.03, *N* = 3). There was suggestive evidence of a difference (Randomization test: *t* = 2.77, df = 4.94, *P* = 0.056, Fig. 2d), again with a large effect (*g* = 1.79, 95% CI = −0.27, 3.85).

#### General Effects: Interaction Behavior

When we assessed immediate effects, on average, juveniles in the experimental condition interacted with the novel box more times (M ± SE = 74.25 ± 14.39, *N* = 4) than juveniles in the control condition (M ± SE = 19.00 ± 12.34, *N* = 3). There was suggestive evidence of a difference (Randomization test: *t* = 2.91, df = 4.99, *P* = 0.085; see Fig. 2e), associated with a large effect (*g* = 1.87, 95% CI = −0.22, 3.87).

When we assessed long-term effects, we observed no significant difference between the number of times juveniles in the experimental condition interacted with the novel box (M ± SE = 66.75 ± 16.63, *N* = 4) compared with juveniles in the control condition (M ± SE = 69.0 ± 23.81, *N* = 3; Randomization test: *t* = −0.08, df = 3.82, *P* = 0.943; see Fig. 2f), with a small effect (*g* = −0.05, 95% CI = −1.70, 1.60).

## Discussion

We found that playback calls seemingly have a direct and immediate impact on the behavior of juveniles towards the box (interaction), their focus on the right part of the box to obtain food (insertion), and their foraging behavior. Although the effect of food-offering calls on approach behavior in juveniles had been observed in previous studies of golden lion tamarins, it had not previously been tested using playbacks (Boinski *et al.* 1994). The effect of food-offering calls on foraging success had also been described in the wild, but never directly tested (Rapaport 2011; Rapaport and Ruiz-Miranda 2002). Our experimental findings show that playback calls seemed to influence the juveniles’ interaction with the box immediately, leading to more insertion and eating events. This may have allowed juveniles to learn the substrates’ food affordances, with the calls attracting them to the substrate.

Although food-elicited calls have not been studied extensively in golden lion tamarins, previous studies found no effects of age (subadult vs. adult), sex, or age of nearest neighbor, and few group differences in the use of calls (Boinski *et al.* 1994). This suggests that golden lion tamarins may use vocalizations as honest signals of their location and activity, as each vocalization is also associated with a specific ecological context. The honesty of the vocalization could help group members coordinate their movement and activity, and facilitate cooperation (Boinski *et al.* 1994). Our data are consistent with these findings and this combination of evidence suggests that food-offering calls are used to attract juveniles to a specific substrate on which it is profitable to forage, and about which they learn.

We also report evidence for longer-term effects of the calls on juvenile learning. We found that, months later, once independent, juveniles in the experimental condition ate more food from the box than subjects that did not receive the playbacks. Our finding that food-offering calls have long-lasting effects on the juveniles’ efficiency at obtaining food from a substrate are consistent with natural observations that food-offering calls potentially teach juveniles about the substrate on which to forage. However, further investigation of the first two criteria of Caro and Hauser’s (1992) is necessary for food-offering calls to be considered teaching. There are only 15 reported cases meeting the first criterion (tutor behavioral modification) in wild golden lion tamarins, and the second criterion (cost of tutor behavior) still requires quantification.

We used playbacks of food-offering calls to mimic a teaching scenario in experiments with wild golden lion tamarins, allowing us to examine whether the learning rate of juveniles was increased by such calls. An alternative causal route for how playback could affect the juveniles’ behavior would be that that they influenced the adults’ behavior, which in turn affected the juveniles’ behavior. However, we found no difference in the foraging behavior of adults in the control and experimental conditions, making it unlikely that the differences between conditions in juvenile behavior arise from differences in adult behavior.

In principle, calls could also influence the probability of success of insertion events, for instance, by enhancing processing skills, or increasing perseverance and motivation to obtain food from the box. We used the proportion of an insertion event leading to an eating event as a measure of perseverance. However, no effects of playback of food-offering calls were found on the probability of success of an insertion event. Seemingly, food-offering calls attracted juveniles to the box, but the pattern of foraging exhibited at that box was seemingly not influenced by the calls. In practice, once individuals had inserted their hand in the box, it was quite easy for them to obtain food. However, success for juveniles plateaued at ca. 70% because of competition for access to the food and resulting displacement.

Prey capture success in golden lion tamarins is highly variable (Miller and Dietz 2005), and foraging success rates vary 15–60% for adults, and are at 87% for juveniles in the 15 cases reported as putative teaching behavior (Dietz *et al.* 1997; Peres 1989; Rapaport 2011; Rapaport and Ruiz-Miranda 2002). Compared to that in adults, the success rate observed in our experiment is slightly higher than those reported previously, which could be because the food we used could not escape the substrate (we used bananas instead of insects for logistical and ethical reasons). Compared to the 15 cases of putative teaching, the success rate in our experiment was slightly lower. It is possible that invitational signals other than, or in combination to, food-offering calls could increase the juveniles’ success.

When tasks are easy to learn, callitrichids often rely on asocial rather than social information (Kendal *et al.* 2009). However, in our study, juveniles still seem to rely on social information despite a success rate of 70%. Another possibility is that callitrichids might rely on social information to learn about substrates, and on asocial information to learn skills such as extracting and manipulating prey. In fact, in our experiment, juveniles were more successful at obtaining food once they had inserted their hands during the period assessing long-term effects than they were in the period assessing immediate effects. This suggests that in the interval between two periods juveniles have 1) become better at competing for access, or less neophobic; 2) learned how to manipulate the box more efficiently through personal experience with that box; or 3) gained manipulative and extractive skills outside the experiment, which they now use in this context.

A potential limitation of the experiment is the use of playback stimuli of calls from individuals that are unknown to the animals that took part. We do not know whether there are individual differences in food-offering calls, whether golden lion tamarins distinguish between familiar and unfamiliar food-offering calls, nor whether the juveniles would behave differently if they did. Adults are quite tolerant of juveniles from other groups: they let juveniles from other groups play with their own juveniles without interfering, and occasionally even let juveniles from other groups take food from them (C. A. Troisi *pers. obs*.). Given this tolerance, it is likely that the familiarity of the calls would not influence the juveniles’ behavior; however, this is an issue for further experimentation.

Future research is required to clarify the effects of age and call type on the juveniles’ behavior. While we cannot rule out the possibility that small differences in the age of experimental and control juveniles affect their foraging behavior, we find this explanation unlikely as ca. 12 days difference at age 4–5 mo, and ca. 6 days difference at age 11–12 mo are unlikely to influence performance. We also cannot rule out the possibility that the production of any type of call supports learning, rather than specifically the food-offering calls. A further control condition where playback of food calls were played would not entirely resolve the problem because 1) food calls would not draw attention to the substrate in exactly the same way as food-offering calls, and 2) food calls do not attract individuals to the food source (Brown and Mack 1978). Moreover, when the analyses conducted in this experiment were carried out on adults no evidence of an effect of the playbacks on learning was found. This suggests that food-offering calls are specifically targeted to encourage juveniles to approach.

We did not directly investigate the social learning mechanisms underlying the juveniles’ learning, but juveniles possibly learn through stimulus enhancement (Heyes 1994; Hoppitt and Laland 2013). In fact, playbacks possibly attract the juveniles to the box where the food is located, enabling a greater opportunity for those juveniles to interact with the source of the food (i.e., the box in our experiment), and extract food from it. This would allow them to learn the association between the box and the presence of food. Stimulus enhancement is a more likely explanation for our findings than local enhancement because the location of the box varied between trials. Future work should investigate the underlying mechanism responsible for the juveniles’ learning, by comparing conditions where the location of the food changes to conditions where it does not.

In cotton-top tamarins, a closely related species, adults use rapidly repeated versions of food calls as food-offering calls, mainly in the presence of infants and juveniles (Dillis *et al.* 2010; Joyce and Snowdon 2007). Adults also transfer food to twins earlier than to singletons, and twins fed independently and used food-associated calls earlier than singletons, supporting the learning hypothesis (Joyce and Snowdon 2007). However, begging and food transfers impede learning, and adult withdrawal of food transfers is needed to encourage independent foraging (Dillis *et al.* 2010; Humle and Snowdon 2008; Joyce and Snowdon 2007). The change of context in the use of food-offering calls in golden lion tamarins, from indication of transfer to indication of food source, could also encourage earlier independent foraging, as our results suggest. Moreover, the use of food-associated calls in a foraging context could also help juveniles learn what food to eat and what vocalization to emit in the presence of food (Roush and Snowdon 2001). In another case of teaching, pied babblers teach their young to associate purr calls with food delivery (Raihani and Ridley 2008). When young become mobile, adults switch the context in which they use the purr calls and emit them to attract fledglings to suitable foraging sites (Radford and Ridley 2006). In this second context, no learning is necessary. It is possible that something similar occurs with the golden lion tamarins. If adults teach their young to associate food-offering calls with food delivery (since those calls are mainly used prior to food transfers), the learning observed in this experiment may be a byproduct of adults attracting juveniles to a good foraging location so that they can obtain nutrients, instead of adults teaching juveniles to forage on specific substrates, as proposed by Rapaport (2011). It would be interesting to investigate how juveniles learn to associate food-offering calls with the presence of food, and whether the adults actively modify their behavior to facilitate their learning, as seen in pied babblers. Comparing the two very similar cases of teaching in golden lion tamarins and pied babblers, which are evolutionary distant species, should help researchers to understand better the constraints under which teaching behavior evolves, and the role that cooperative breeding might play in the evolution of this behavior.

## Conclusion

We found suggestive evidence that food-offering calls in golden lion tamarins facilitate learning of which substrates to forage on. Our findings are consistent with Rapaport’s teaching hypothesis (2011). Rapaport initially suggested that as juveniles grow older, adults start using food-offering calls to direct the juveniles’ attention towards a particularly profitable substrate. Lion tamarins have a high reproductive turnover strategy: they show intense parental investment, have multiple births per year of twins, and mature rapidly (Brown and Mack 1978). In anthropoid lineages, the high success of survival and reproduction is in part due to learning occurring during a long maturation period. Teaching could therefore be a strategy to speed up the learning of valuable skills and information, and thereby reduce the burden of provisioning by hastening the transition to independent foraging. However, the question remains as to whether the learning observed in this experiment is the function of the calls (i.e., teaching) or whether it is a byproduct of (allo-) parental care. Further experiments investigating the contexts in which individuals teach will enable researchers to better understand the evolution of this costly behavior.

## Electronic supplementary material

ESM 1 (DOCX 109 kb)
