# Supplementary material for: Food-Offering Calls in Wild Golden Lion Tamarins (Leontopithecus rosalia): Evidence for Teaching Behavior?
Source: Int J Primatol. 2018 Nov 21;39(6):1105–23. doi: 10.1007/s10764-018-0069-z (PMC6300579; doi:10.1007/s10764-018-0069-z)
Supplement: Supplementary file 1 — (DOCX 109 kb) [file 10764_2018_69_MOESM1_ESM.docx]

**Electronic Supplementary Material**

**Food-Offering Calls in Wild Golden Lion Tamarins (*Leontopithecus rosalia*): Evidence for Teaching Behavior?**

**Camille A. Troisi · Will J. E. Hoppitt · Carlos R. Ruiz-Miranda · Kevin N. Laland**

**Table SI** List of individuals that took part in the experiment. Ad = adult; Sub = subadult; Juv = juvenile; inf = infant; ABS = absent. Note that data of infants were not analyzed.

| Locality | Group | Individual | Sex | Date of birth | Age when immediate effects were assessed | Age when long-term effects were assessed | Present  when immediate effects were assessed | Present when long-term effects were assessed | Condition | Start date when immediate effects were assessed | Start date when long-term effects were assessed |
| --- | --- | --- | --- | --- | --- | --- | --- | --- | --- | --- | --- |
| Poco | BO2 | 1284 | M | — | Ad | Ad | ✓ | ✓ | Ctl | 15/03/2014 | 13/09/2014 |
| Poco | BO2 | 1278 | F | — | Ad | Ad | ✓ | ✓ | Ctl | 15/03/2014 | 13/09/2014 |
| Poco | BO2 | 1342 | M | 10/12 | Sub | Ad | ✓ | ✓ | Ctl | 15/03/2014 | 13/09/2014 |
| Poco | BO2 | 1343 | M | 10/12 | Sub | Ad | ✓ | ✓ | Ctl | 15/03/2014 | 13/09/2014 |
| Poco | BO2 | 1351 | M | 02/13 | Sub | Ad | ✓ | ✓ | Ctl | 15/03/2014 | 13/09/2014 |
| Poco | BO2 | 1352 | F | 02/13 | Sub | Ad | ✓ | ✓ | Ctl | 15/03/2014 | 13/09/2014 |
| Poco | BO2 | 1353 | M | 09/13 | Juv | Sub | ✓ | ✓ | Ctl | 15/03/2014 | 13/09/2014 |
| Poco | BO2 | 1354 | F | 09/13 | Juv | Sub | ✓ | ✓ | Ctl | 15/03/2014 | 13/09/2014 |
| Poco | AF | FA3 | M | 10/09 | Ad | Ad | ✓ | ✓ | Ctl | 9/03/2014 | 7/09/2014 |
| Poco | AF | AF13 | F | 12/08 | Ad | Ad | ✓ | ✓ | Ctl | 9/03/2014 | 7/09/2014 |
| Poco | AF | AF19 | M | 10/11 | Ad | ABS | ✓ |  | Ctl | 9/03/2014 | 7/09/2014 |
| Poco | AF | AF20 | F | 12/11 | Ad | Ad | ✓ | ✓ | Ctl | 9/03/2014 | 7/09/2014 |
| Poco | AF | AF35 | M | 12/12 | Sub | Ad | ✓ | ✓ | Ctl | 9/03/2014 | 7/09/2014 |
| Poco | AF | AF27 | F | 10/13 | Juv | Sub | ✓ | ✓ | Ctl | 9/03/2014 | 7/09/2014 |
| Poco | Alone | 1303 | M | — | Ad | Ad | ✓ | ✓ | Exp | 15/03/2014 | 9/09/2014 |
| Poco | Alone | 1313 | F | 11/09 | Ad | ABS | ✓ |  | Exp | 15/03/2014 | 9/09/2014 |
| Poco | Alone | 1360 | F | — | ABS | Ad |  | ✓ | Exp | 15/03/2014 | 9/09/2014 |
| Poco | Alone | 1355 | M | 10/13 | Juv | Sub | ✓ | ✓ | Exp | 15/03/2014 | 9/09/2014 |
| Poco | Alone | 1356 | M | 10/13 | Juv | ABS | ✓ |  | Exp | 15/03/2014 | 9/09/2014 |
| Afetiva | AF3 | PT8 | M | 10/01 | Ad | Ad | ✓ | ✓ | Exp | 24/02/2014 | 20/09/2014 |
| Afetiva | AF3 | SP18 | M | 10/09 | Ad | Ad | ✓ | ✓ | Exp | 24/02/2014 | 20/09/2014 |
| Afetiva | AF3 | FA2 | F | 12/08 | Ad | Ad | ✓ | ✓ | Exp | 24/02/2014 | 20/09/2014 |
| Afetiva | AF3 | FP1 | M | 12/12 | Sub | ABS | ✓ |  | Exp | 24/02/2014 | 20/09/2014 |
| Afetiva | AF3 | FP2 | F | 12/12 | Sub | Ad | ✓ | ✓ | Exp | 24/02/2014 | 20/09/2014 |
| Afetiva | AF3 | FP3 | F | 09/13 | Juv | Sub | ✓ | ✓ | Exp | 24/02/2014 | 20/09/2014 |
| Afetiva | AF3 | FP4 | M | 09/13 | Juv | Sub | ✓ | ✓ | Exp | 24/02/2014 | 20/09/2014 |
| Afetiva | Super | SP16 | F | 11/08 | Ad | Ad | ✓ | ✓ | Exp | 19/02/2014 | 20/09/2014 |
| Afetiva | Super | FA4 | M | 10/09 | Ad | Ad | ✓ | ✓ | Exp | 19/02/2014 | 20/09/2014 |
| Afetiva | Super | SP20 | M | 11/11 | Ad | Ad | ✓ | ✓ | Exp | 19/02/2014 | 20/09/2014 |
| Afetiva | Super | SP23 | F | 12/12 | Sub | Ad | ✓ | ✓ | Exp | 19/02/2014 | 20/09/2014 |
| Afetiva | Super | SP24 | F | 12/12 | Sub | Ad | ✓ | ✓ | Exp | 19/02/2014 | 20/09/2014 |
| Afetiva | Super | SP25 | F | 12/12 | Sub | ABS | ✓ |  | Exp | 19/02/2014 | 20/09/2014 |
| Afetiva | Super | SP26 | F | 09/13 | Juv | Sub | ✓ | ✓ | Exp | 19/02/2014 | 20/09/2014 |
| Afetiva | Super | SP27 | F | 02/14 | Inf | Juv | ✓ | ✓ | Exp | 19/02/2014 | 20/09/2014 |
| Afetiva | Super | SP28 | F | 02/14 | Inf | Juv | ✓ | ✓ | Exp | 19/02/2014 | 20/09/2014 |

**Table SII** Results of randomization tests testing for difference in juvenile golden lion tamarins’ behavior in groups that were provided with playbacks (experimental condition) and in groups that were not (control condition). Only the results showing suggestive evidence of a difference between the behavior of juveniles in the control and experimental groups (0.05 < *P* < 0.1) are shown, with the 90% CI. Poco das Antas and Affetiva, February to March 2014 (immediate effects) and September to October 2014 (long-term effects)

| Behavior | Period | *T* | df | *P*-value | *g* | 95% CI | 90% CI |
| --- | --- | --- | --- | --- | --- | --- | --- |
| Insertion | Immediate effects | 2.34 | 3.61 | 0.087 | 1.50 | –0.45, 3.45 | –0.03, 3.03 |
| Eating | Immediate effects | 2.41 | 4.39 | 0.057 | 1.55 | –0.42, 3.52 | 0.01, 3.09 |
| Eating | Long-term effects | 2.77 | 4.94 | 0.056 | 1.79 | –0.27, 3.85 | 0.17, 3.40 |
| Interaction | Immediate effects | 2.91 | 4.99 | 0.085 | 1.87 | –0.22, 3.87 | 0.23, 3.52 |

*g* refers to the effect size.

**
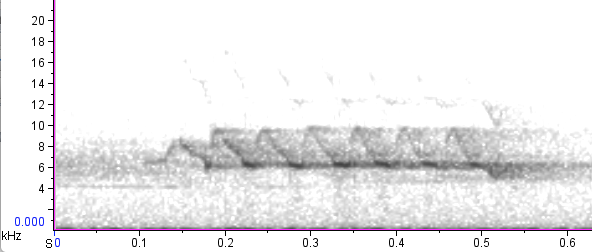
**

**Fig. S1** Spectrogram of one of the food-offering calls used in the experiment.
